# Stage-Wise Curing for Improving the Bonding Strength of Imaging Coupling Devices

**DOI:** 10.3390/ma19081562

**Published:** 2026-04-14

**Authors:** Yuwen Xing, Yajie Du, Miao Chu, Peng Jiao, Yang Fu, Zeping Sun, Miao Dong, Yonggang Huang

**Affiliations:** 1 Institute of Special Glass Fiber, China Building Materials Academy, Beijing 100024, China; xyw_0221@163.com (Y.X.); chumiaobit@163.com (M.C.); fuyang0517@126.com (Y.F.); dongmiao@cbma.com.cn (M.D.); 2 Key Laboratory of Special Optoelectronic Glass in Building Materials Industry, China Building Materials Academy, Beijing 100024, China

**Keywords:** ICCD/ICMOS, UV curing adhesive, oxygen blocking aggregation effect, imaging coupling device, stage-wise curing

## Abstract

In extreme scenarios such as nuclear explosions and high-energy radiation detection in space, UV-cured adhesives are usually used as coupling media to bind tapered optic fiber arrays with intensified charge-coupled devices or complementary metal–oxide semiconductors and a tapered optic fiber array for effective optical signal transmission. To address the issue of weak bonding strength caused by the small binding area between charge-coupled devices or complementary metal–oxide semiconductors and TOFA, a stage-wise curing process was investigated and proved to be efficient through comparison with the single curing process. The effect of interval time between the initial and final curing on coupling strength was characterized by tensile strength, shear strength and shock acceleration testing, and the samples were exposed to high and low temperatures for evaluation of their environmental adaptability. The curing mechanism was analyzed by surface morphology of the adhesive layer after decoupling and an energy-dispersive X-ray spectroscopy elemental analysis of interface layer. The results show that when the interval time is extended from 5 min to 60 min, the shock acceleration of the coupling device decreases by 26.1%, while the tensile and shear strengths also decrease by 49.4% and 60.7%, respectively. The decline in coupling strength is attributed to oxygen inhibition during interval time. The exposure of the adhesive surface to the air allows oxygen to diffuse into and react with active the free radicals that remain from the initial curing, which inhibits further polymerization and generates a thin, incompletely cured weak boundary layer. These findings provide insights for optimizing stage-wise curing processes and improving the reliability of coupled imaging devices.

## 1. Introduction

When the detective equipment is applied in some extreme scenarios, including nuclear explosion diagnostics, inertial confinement fusion, and cosmic high-energy radiation detection, the efficient conversion and acquisition of weak light and high-energy radiation signals are largely dependent on intensified charge-coupled devices (ICCDs) and intensified complementary metal–oxide semiconductors (ICMOSs). The core structure of ICCDs and ICMOSs is similar, consisting of the “image intensifier–tapered optic fiber array–CCD/CMOS” combination [1,2,3,4,5]. The tapered optic fiber array (TOFA) is coupled with the image intensifier at the input end and closely bonded to the photosensitive surface of the CCD/CMOS chip at the output end. This allows it to serve as a means of both image magnification/reduction and optical signal transmission. The intensified optical image could be completely transferred to the chip’s photosensitive surface while minimizing optical signal loss [6,7,8,9,10,11]. Therefore, the coupling between TOFA and CCD/CMOS directly determines the ultimate performance of detective devices. Any delamination or debonding in the interface between TOFA and CCD/CMOS would decrease transmission efficiency and distort images. This may lead to complete device failure when operating under extreme conditions.

Reliable coupling between TOFA and CCD/CMOS requires an optical adhesive that combines high transparency with good bonding strength [12,13]. There are various curing processes available, such as the thermal curing of polyvinyl alcohol (PVA) or epoxy resins, which are widely used in electronic packaging [14,15]. UV-cured adhesives have been the mainstream coupling medium in this field due to a high visible light transmittance exceeding 90%, fast curing, and excellent bonding properties [16,17,18]. There are some studies about UV curing. Scherzer et al. indicated that the oxygen concentration not only reduces the degree of surface polymerization but also affects the formation of the overall cross-linked network during the UV curing process [19]. This results in a decrease in final bonding strength. Studer et al. further quantified that during UV curing in-air, the polymerization rate is only one-quarter of that achieved under an inert atmosphere, with a significant reduction in the final conversion rate [20]. The investigation into dark polymerization from Johnson et al. also noted that changes in the chemical state during the curing interval significantly influence the final degree of polymerization [21]. It is worth noting that the susceptibility to oxygen inhibition varies with the chemical system of the adhesive. For instance, thiol–ene systems exhibit lower oxygen sensitivity due to their distinct radical step-growth mechanism [22]. Furthermore, studies have explored free radical/cationic hybrid UV-cured systems in which silyl radicals effectively reduce the oxygen inhibition effect [23]. For the coupling of TOFA with CCD/CMOS chips, achieving sufficient bonding strength with a single curing stage is challenging due to the small chip size. The limited bonding area reduces the total load-bearing capacity, as the maximum load that the interface can withstand is directly proportional to the bonding area. Consequently, a smaller bonding area leads to a lower maximum load that can be supported before failure occurs, meaning a single curing stage is insufficient to meet reliability requirements. A stage-wise curing is usually employed to enhance coupling reliability. Our research team has developed a two-stage curing to successfully provide an end-product for application. However, improving the coupling stability is still a challenge that requires in-depth research on the influence of the stage-wise curing process on the coupling strength. Such findings are valuable and necessary for guiding practice to achieve highly reliable TOFA–CCD/CMOS coupling.

This study systematically investigated the effect of the stage-wise curing process on coupling strength. The evaluation involved tensile strength, shear strength, shock acceleration resistance, and environmental adaptability to high and low temperatures. To elucidate the underlying mechanism, the interfacial microstructure was analyzed in combination with surface chemical-state characterization. This multi-scale approach reveals how the two-stage process alters the adhesive layer’s surface state and, ultimately, its bonding performance—from macroscopic environmental adaptability to microscopic interface structure. The evolution of the interface during the two-stage process was identified, and controlling the interval time between the two curing stages to within 10 min was demonstrated to significantly enhance coupling strength.

## 2. Experiment

### 2.1. Coupling Process

**Figure 1:**
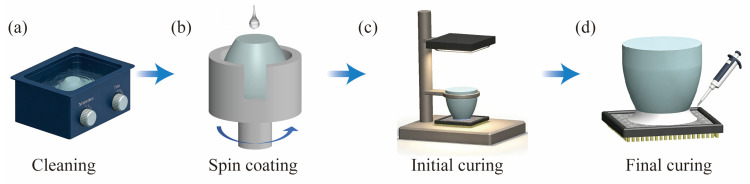
Schematic diagram of the coupling process: (**a**) cleaning the tapered fiber array; (**b**) spin-coating the UV adhesive; (**c**) initial curing stage; (**d**) final curing stage.

A schematic diagram of the coupling process is shown in Figure 1. The TOFA was sequentially ultrasonically cleaned in deionized water, anhydrous ethanol, and acetone for 30 min each. After cleaning, it was dried in an oven at 60 °C for 30 min to remove surface oil and impurities. UV adhesive was spin-coated onto the clean TOFA, with the layer thickness controlled within 10 μm. The UV adhesive used in this study is an acrylate-based photosensitive resin (H834, QUINSON, Guangzhou, China), which exhibits high optical transparency (>90% in the visible light range) and good adhesion properties. The TOFA was aligned with the CCD chip using a high-precision positioning stage, ensuring the coaxial error was less than 0.05 mm. After confirming that the coupling interface was free of bubbles, moiré patterns, and Newton’s rings, UV curing was initiated by irradiation for 10 min to complete the initial curing stage. Approximately 0.1 mL of UV adhesive was dispensed around the junction between the TOFA and the CMOS using a micropipette, ensuring complete coverage without overflow. The secondary curing stage was completed by UV irradiation for another 5 min.

### 2.2. Measurement and Characterization

#### 2.2.1. Bonding Strength

The tensile tests were performed using a Zwick Roell-Z020 universal testing machine (Zwick Roell, Ulm, Germany). The samples were secured in the test fixture, and the tensile load was ramped from zero until the sample detached from the CMOS. The shear tests were conducted using a Zwick Roell-Z020 universal testing machine (Zwick Roell, Ulm, Germany). Samples were secured in the test fixture, and shear load was ramped from zero until the sample detached from the CMOS. The shock tests were performed using a KRD11-100 pneumatic impact test machine (KRD, Shanghai, China). The impact level was incrementally increased from 10 g with a pulse width of 11 ms until the sample detached from the CMOS. It should be noted that all mechanical test samples failed under the corresponding applied loads, and the critical load values at failure were recorded.

The environmental adaptability was tested using a Hongjin HYE-TH-80DH programmable temperature and humidity chamber (Hongjin, Dongguan, China). A charge sample was placed inside the chamber and experienced increasing and decreasing temperatures compared to the target one. After maintaining these for 1.5 h, images were captured to observe whether the camera functioned normally and the mechanical properties were measured.

#### 2.2.2. Microstructure Characterization

Optical images were acquired using an optical microscope (JINTUOFENGYIQI, Beijing, China) equipped with a 10× eyepiece and 18× objective, providing a total magnification of 180×.

SEM images were obtained using a ZEISS-Sigma 500 field-emission scanning electron microscope (FE-SEM) (ZEISS, Oberkochen, Germany) at an accelerating voltage of 5 kV.

Elemental composition analysis was performed using an energy-dispersive X-ray spectroscope (EDS) attached to the ZEISS-Sigma 500 SEM, operating at an accelerating voltage of 15 kV.

Raman spectra were acquired using a DXR2xi Raman spectrometer (Thermo Fisher Scientific, Waltham, MA, USA) over a spectral range of 400–3000 cm^−1^.

## 3. Results and Discussion

**Figure 2:**
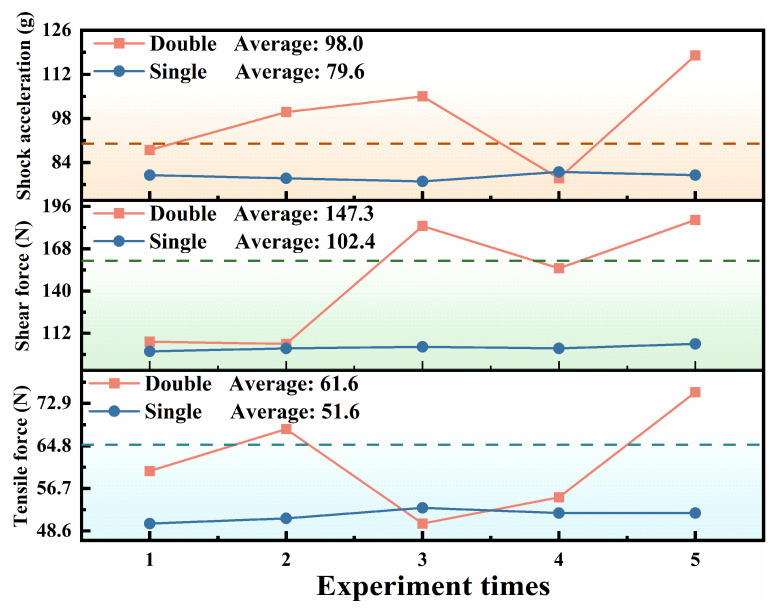
Tests for tensile strength, shear strength and impact velocity after single and double curing.

Due to the small coupling area between TOFA and CMOS, the thickness of the adhesive layer was controlled within 10 μm. To further enhance the bonding strength, coupling adhesive was applied around the interface after the initial curing stage, followed by the final curing stage. All cured samples’ including tensile and shear strength, and their shock acceleration, were measured. To clarify the strength difference between single and double curing processes, comparative mechanical performance tests were conducted and are shown in Figure 2. Using single curing, the average tensile strength (51.6 N), shear strength (102.4 N), and shock acceleration (79.6 g) of the samples all failed to meet the required values (indicated by the dashed line in Figure 2). Although the stage-wise curing process improves the average values of all mechanical strength, the measured results exhibit significantly increased fluctuation, indicating insufficient process stability.

**Figure 3:**
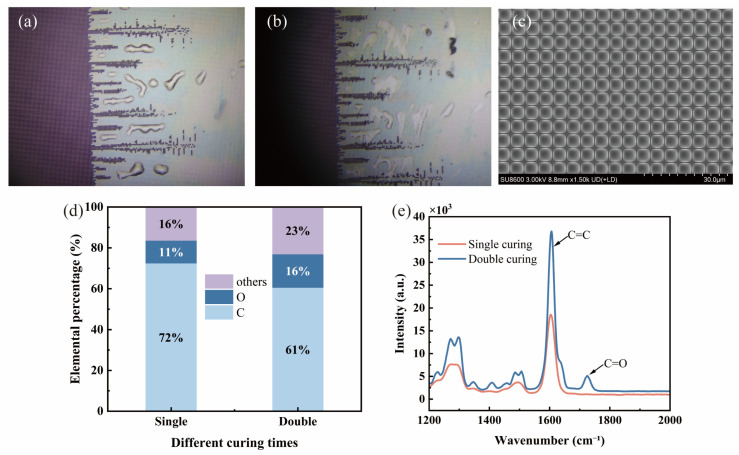
Structural characterization of qualified and unqualified samples: (**a**,**b**) optical microscope images of unqualified samples after decoupling; (**c**) surface morphology of the adhesive layer after decoupling; (**d**) EDS elemental analysis of the sample interface layer; (**e**) Raman spectroscopy comparison.

To further clarify the reasons for the performance fluctuations in the stage-wise cured samples, a structural characterization was performed on both qualified and unqualified samples. After the coupled device is decoupled, the surface of unqualified samples reveals a layer of liquid material that is wipeable but resistant to solidification under subsequent UV irradiation (as shown in Figure 3a,b). Figure 3c shows the surface morphology of the unqualified sample after decoupling. Due to interface damage during the decoupling process, the adhesive layer surface is no longer smooth, with imprints of the chip structure remaining in local areas. EDS elemental analysis results indicate that, compared to qualified samples, the carbon element content in the interface region of the unqualified samples is significantly reduced while the oxygen element content is obviously elevated (as shown in Figure 3d). Figure 3e shows a Raman spectroscopy comparison between the qualified and unqualified samples. In the Raman spectrum of the unqualified sample, it is seen that the intensities of both the C=C stretching vibration peak at 1640 cm^−1^ and the C=O stretching vibration peak at 1720 cm^−1^ are significantly higher than those of the qualified sample. The presence of C=O and the increased intensity of the C=C peak indicates incomplete curing. This result is consistent with the EDS elemental analysis. The above results indicate the presence of a chemically inactive weak boundary layer at the interface of the unqualified samples. This structural defect directly affects the macroscopic mechanical properties of the device, including tensile, shear, and impact resistance.

**Figure 4:**
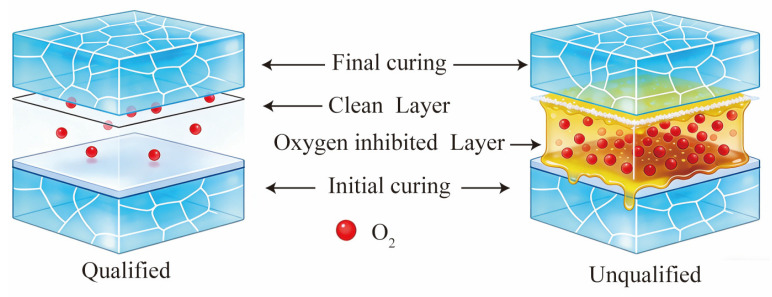
Schematic diagram of oxygen inhibition effect during the final curing stage.

Figure 4 presents a schematic diagram of the interface structure after stage-wise curing for qualified and unqualified samples. For the qualified sample, the adhesive layer surface is smooth after the initial curing stage, and some active free radicals are retained. When the adhesive is applied in the final stage, a clean interface is formed between the two adhesive layers. During the final curing, the monomers in the new layer could react directly with the active sites on the surface, achieving chemical bonding and resulting in high interfacial strength. In the case of the unqualified sample, a viscous liquid layer is formed on the adhesive surface after the initial curing stage. This layer, mainly composed of unreacted monomers, oligomers, and oxidation products, could not be cured by further UV irradiation. When the adhesive is applied in the final stage, this viscous layer prevents direct contact between the two adhesive layers, forming a weak boundary interface. This leads to structural defects at the interface after the final curing, preventing the achievement of the expected coupling strength. This conforms to the oxygen inhibition effect. During the UV adhesive curing process, oxygen molecules preferentially react with active free radicals (*R*•) to generate low-activity peroxyl radicals (*ROO*•) (Equation (1)). Although *ROO*• itself can hardly initiate monomer polymerization, it can react with residual *R*• in the system to form stable, inactive products (Equation (2)). The reduced reactivity of peroxyl radicals (*ROO*•) following monomer addition is a well-established concept in radical polymerization chemistry. Compared to carbon-centered radicals (*R*•), peroxyl radicals are stabilized by resonance and exhibit lower exothermicity in propagation reactions, making them less effective at initiating or sustaining polymerization [20].

(1) $$R•+O_{2}⟶\;ROO$$

(2) ROO•+R• ⟶Inactive product

During curing, the oxygen content at the interface is governed solely by the interval time. The interval between the initial and final curing stages directly determines the extent of this chemical state’s evolution. Therefore, this research systematically investigates the influence of different intervals between curing stages on the coupling strength. Seven groups of control samples were prepared with different intervals. After reaching the set interval time for each group, adhesive was applied again at the interface between the TOFA and the chip, and the final curing stage was completed by UV irradiation for another 5 min.

**Figure 5:**
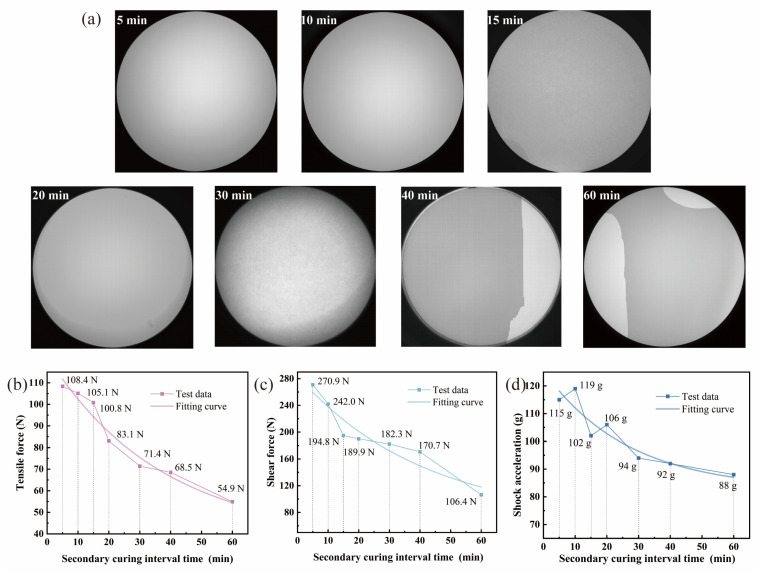
Environmental adaptability tests with different intervals between two curing stages: (**a**) camera function; (**b**) tensile strength; (**c**) shear strength; (**d**) shock acceleration.

Environmental adaptability is one of the key indicators when evaluating the performance of the coupled assembly in extreme scenarios. Therefore, the samples were charged and operated for 1.5 h at temperatures of both 5 °C and 40 °C, and the camera function results are shown in Figure 5a. If the grayscale values fall within the circular effective area, the image is deemed normal and the sample is qualified. For samples with 5 min and 10 min intervals, grayscale values remain uniform after 1.5 h operation at both temperatures, with no signs of debonding. In contrast, for the other five groups of samples, with intervals of 15 min or longer, significant differences in grayscale values appear within the circular effective area. This indicates that an interfacial bonding state occurs, with debonding occurring in local areas. As the interval time increases, the area with grayscale differences spreads and the debonding phenomenon is intensified. In addition to static temperature exposure, thermal cycling may further compromise the bonding interface when the interval time is long. The mismatch in the coefficients of thermal expansion between the weak boundary layer and the fully cured adhesive layer generates cyclic stresses, which can accelerate interfacial debonding. This highlights the importance of minimizing the weak boundary layer in devices intended for operation under varying temperature conditions. These results demonstrate that the interval between the two curing stages has a significant impact on the environmental adaptability of the device.

Figure 5b–d present the mechanical performance of devices with different intervals between the stage-wise curing. The tensile strength is shown in Figure 5b. At an interval of 5 min, the critical tensile strength reaches a peak of 108.4 N, indicating complete polymerization of the adhesive layer and a tightly bonded interface. As the interval time increases, the critical tensile strength shows a continuous downward trend from 105.1 N at 10 min (a 3.0% decrease compared to 5 min) to 71.4 N (a 34.1% decrease) at 40 min. At 60 min, it is only 54.9 N (a decrease of 49.4%). The tensile load is vertically applied to the coupling interface, so any change in the surface state caused by oxygen inhibition directly weakens the interfacial bonding force. With a short interval (less than 10 min), a high concentration of active free radicals remains on the surface layer of the adhesive after the initial curing. The final curing can effectively reactivate these residual radicals, promoting the continuous growth of the polymer chains. The layer affected by oxygen inhibition is extremely thin, having a limited impact on the critical tensile strength. As the interval extends, the reaction between oxygen and free radicals continues, and the surface radicals gradually deactivate. The final curing fails to effectively polymerize this layer. The oxygen-inhibited layer thickens, forming a stress concentration zone. This leads to interfacial debonding at a lower tensile load.

The shear strength is shown in Figure 5c. At an interval of 5 min, the critical shear strength reaches a peak of 270.9 N, indicating complete polymerization of the adhesive layer and strong interfacial bonding. As the interval time increases, the critical shear strength continuously decreases. The shear load is parallel with the coupling interface. The shear modulus of the layer affected by oxygen inhibition is much lower than that of the fully cured adhesive (by approximately one third), rendering it susceptible to failure under low shear loads. With a short interval (less than 10 min), the fully cured adhesive layer dissipates most of the shear load, and the oxygen-inhibited layer has a negligible impact on strength. With an interval exceeding 20 min, the oxygen-inhibited layer becomes the primary failure zone under shear load, causing interfacial debonding failure of the sample under a lower shear load.

The shock acceleration is shown in Figure 5d. Consistent with the tensile and shear tests, a shorter interval results in the sample being able to withstand higher critical acceleration before impact failure. At an interval of 5 min, the critical shock acceleration reaches a peak of 115 g. The adhesive layer is tightly bonded to the components, effectively absorbing impact energy, and an acceleration of 115 g is required to initiate interface delamination. As the interval time increases, the critical shear strength continuously decreases. The duration of the impact load is extremely short. Part of the impact energy can be absorbed by the bulk structure of the TOFA and the CCD chip, thereby delaying the failure of the weak boundary layer. Consequently, critical shock acceleration is less sensitive to interval length than tensile and shear strength. However, with a long interval of over 30 min, the elastic modulus of the layer affected by oxygen inhibition is only one third to half that of the fully cured adhesive layer. This is not enough to effectively buffer the impact stress, and leads to a significant decrease in critical shock acceleration. Combined with the previous interface characterization results, the decline in mechanical properties with extended intervals closely corresponds to the thickening of the weak boundary layer. Comprehensive mechanical performance test results indicate that the interval between the two curing stages has a decisive influence on the device’s tensile, shear, and impact strength. All these results are attributed to the formation of a weak boundary layer at the interface of samples with long intervals due to the oxygen inhibition effect.

## 4. Conclusions

This study identified the key factors governing coupling strength in the stage-wise curing process and systematically investigated how the interval between curing stages affects the performance of ICCD/ICMOS devices. The results showed that when the interval is 10 min or less, all samples are qualified during environmental tests at 5 °C and 40 °C for 1.5 h. At intervals of 15 min or longer, debonding appears and intensifies as the interval increases. Mechanical properties continuously decrease with increasing interval time. As the interval was extended from 5 to 60 min, tensile strength decreased by 49.4%, shear strength decreased by 60.7%, and critical shock acceleration decreased by 26.1%. Interface analysis related this degradation to the oxygen inhibition effect. The prolonged intervals initiate oxygen inhibition, forming an uncured viscous layer on the adhesive surface with retained residual C=C double bonds. The oxygen-enriched surface sharply reduces chemical activity, and ultimately leads to interfacial failure due to stress concentration in this zone. Controlling the interval between curing stages to within 10 min was confirmed to meet the application requirements. This research provides experimental data and theoretical guidance for the process optimization of ICCD/ICCMOS coupling devices and strength control of similar optical coupling devices.

## Data Availability

The original contributions presented in this study are included in the article. Further inquiries can be directed to the corresponding authors.
